# The potential human health hazard of nitrates in drinking water: a media discourse analysis in a high-income country

**DOI:** 10.1186/s12940-023-00960-5

**Published:** 2023-01-20

**Authors:** Leah Grout, Tim Chambers, Simon Hales, Marnie Prickett, Michael G. Baker, Nick Wilson

**Affiliations:** 1 Department of Public Health, University of Otago, Wellington, 6021 New Zealand; 2 Larner College of Medicine, University of Vermont, VT Burlington, USA

**Keywords:** Nitrate, Water, Colorectal cancer, Media, Newspapers, Communication, New Zealand

## Abstract

**Background:**

Recent studies linking low levels of nitrate in drinking water to colorectal cancer have raised public concerns over nitrate contamination. The aim of this study was to analyze the media discourse on the potential human health hazard of nitrates in drinking water in a high-income country with a large livestock industry: New Zealand (NZ).

**Methods:**

Searches of media sources (“major newspapers”) held by the Factiva database for the NZ setting in the five-year period 17 December 2016 to 20 December 2021.

**Results:**

The largest number of media items was observed for 2017 (*n* = 108), the year of a NZ general election, with a notable decrease in 2020 (*n* = 20) that was likely due to the Covid-19 pandemic, which dominated health media. However, the percentage of these media items with a health focus steadily increased over time, from 11.1% of all articles in 2017 to 51.2% in 2021. The most commonly mentioned health hazard was colorectal cancer, followed by methemoglobinemia. The temporal pattern of media items suggests that the release of scientific studies and scholarly blogs was associated with the publication of subsequent media items. Major stakeholders involved in the discourse included representatives of local and central government, environmental and recreational interest groups, researchers, local residents, agricultural interest groups, and health organizations. Māori (Indigenous New Zealanders) values or perspectives were rarely mentioned.

**Conclusions:**

Analysis of major newspapers for a five-year period indicated that a wide range of expert comment and opinions were made available to the public and policy makers on the issue of nitrates in water. While many different stakeholder views were captured in the media discourse, there is scope for the media to better report the views of Māori on this topic. There is also a need for articles detailing the health issues to also refer to the environmental, recreational, and cultural aspects of protecting water quality to ensure that the public, policy makers, and regulators are aware of co-benefits.

**Supplementary Information:**

The online version contains supplementary material available at 10.1186/s12940-023-00960-5.

## Background

Globally, nitrate levels in source water have increased as a result of agricultural intensification [1]. Nitrate is also a common drinking water contaminant in the high-income country of New Zealand (NZ), typically driven by agricultural activities (ie, through the application of nitrogenous fertilizers and livestock urine) [2, 3]. While nitrate levels in most drinking water sources in NZ are far lower than in countries with long traditions of agricultural intensification [1, 4], nitrate levels are gradually increasing to levels that are potentially concerning for ecological and human health. Indeed, nitrate concentrations in many river reaches and lakes in NZ are already at levels that are ecologically damaging [5].

The risk of methemoglobinemia from nitrate intake, particularly in infants, has been well established [6–8]. Recent studies also suggest a link between nitrate exposure during pregnancy and poor birth outcomes, including preterm births [9], low birth weights [10], and congenital anomalies [11]. Emerging evidence also suggests a potential link between nitrate in drinking water and colorectal cancer [12, 13]. NZ has one of the highest colorectal cancer rates in the world, and it is the second highest cause of cancer death in the country [14]. The International Agency for Research on Cancer (IARC) has concluded that ingested nitrate or nitrite, under conditions that result in endogenous nitrosation in the gastrointestinal tract, is likely carcinogenic to humans [15].

The World Health Organization (WHO) has set drinking water standard at 11.3 mg/L to protect against methemoglobinemia [16], which has been adopted in NZ’s drinking water standards [17]. Nevertheless, evidence suggests the risk of preterm birth increases by 47% when nitrate levels are above 5 mg/L [9], and the risk of colorectal cancer increases by 11% when nitrate levels are above 0.87 mg/L [12]. Further, recent genetic and experimental evidence supports epidemiological evidence for a potential link between nitrate and colorectal cancer [18]. Levels of nitrate in NZ drinking water, particularly from unreticulated groundwater supplies is an ongoing concern. A recent study estimated that as many as 138,000 people (3% of the population) in NZ could be using water supplies with nitrate levels above 5 mg/L, while 800,000 (17%) were exposed to greater than 1 mg/L [4]. Conventional water treatment methods are not effective for the reduction of nitrate levels in drinking water [19]. Several other methods such as ion-exchange and reverse osmosis can remove nitrates from drinking water [19], but they may be costly or difficult to implement and operate.

The media plays an important role in communicating health messages to the public. Informed public discourse is needed in domains such as nitrate water pollution where regulatory levels need to be set by considering the risks to health, risks to the environment, cultural impacts, and the impacts on recreational water use (swimming and fishing). Achievement of regulatory levels must also consider the impacts on the agricultural sector. Two previous studies have presented the results of discourse network analyses on the case of nitrate pollution of groundwater. Vogeler et al. [20] examined the public discourse in a region of Germany with intensive livestock farming, while Schaub et al. [21] analyzed newspaper articles and press releases from 2010 to 2020 in Germany to examine discourse over the issue of fertilizer regulation. However, to our knowledge, no media discourse analyses of the issue of nitrates in water have been conducted outside of Europe or specifically focused on the potential health hazard associated with nitrate contamination.

Recent freshwater legislation in NZ has strengthened the framework of *Te Mana o Te Wai,* which addresses the vital importance of water [22]. In practical terms, NZ regulators must now consider a hierarchy of priorities in their decision making on freshwater: (i) the health of the water; then (ii) the use of water for health (eg, drinking water or domestic use); and then (iii) the use of water for economic benefit [23]. Nitrate contamination is of concern for these priorities. Therefore, to assess the nature of the media discourse, we examined media items in NZ’s major newspapers. In particular, we aimed to determine how the release of journal articles, reports and scholarly blogs have influenced subsequent media discourse on nitrates in drinking water in NZ. We also aimed to identify stakeholders and their roles in advocacy and influencing the national and regional policy agenda.

## Methods

We searched the Factiva database for the five-year period preceding 20 December 2021 for media items in major NZ newspapers containing the terms “nitrates” or “nitrate”, and “water”. Media items were identified in five different major newspapers (Supplementary Table 1). According to Roy Morgan company, the combined readership for those newspapers (ie, total cross-platform audience including print, internet, or app) was over three million (Supplementary Table 1) out of a national population of around five million, although there is overlap between the readers of each newspaper. Most of the NZ population is served by the four large metropolitan dailies; the *NZ Herald* and the *Dominion Post* in the North Island, and *The Press* and the *Otago Daily Times* in the South Island, while the *Sunday Star-Times* is a newspaper published each weekend in the largest city: Auckland.

Articles were excluded if they were not directly related to nitrates in freshwater. All syndicated articles were included in the analysis because they reach different audiences and their inclusion helps to better capture the total impact of the media discourse on the public and policy makers.

Included media items were subjected to content analysis. Briefly, codes included reference to potential health hazard, geography of waterbodies, and stakeholders mentioned. The full coding schedule is available in Supplementary Table 2.

### Assessment of inter-observer variation

Using a random number generator, we took a random 10% sample of media items that had been coded by the first author and another author coded these independently. Then a third author compared the findings and analyzed for inter-observer variation. This was calculated as per Cohen’s kappa [24] for the ten most important data items. Interpretation of the kappa scores was as per Landis and Koch [25]: 0.21 – 0.40 as “fair agreement”, 0.41 – 0.60 as “moderate agreement”, 0.61 – 0.80 as “substantial agreement”, and 0.81 – 1.00 as “almost perfect or perfect agreement”.

## Results

From 17 December 2016 to 20 December 2021, 346 media items were published in the selected major NZ newspapers that mentioned the terms “nitrate” or “nitrates” and “water”. Of those, 29 were excluded because they were not related to nitrates in freshwater. The remaining 317 media items were analyzed for content. Twenty-seven (8.5%) of the included media items were syndicated (ie, they were largely similar or identical to another media item that appeared in another newspaper).

The random 10% sample of media items analyzed for inter-observer variation gave agreement levels of 75% to 100% (mean = 89%, median = 92%), with Cohen’s kappa scores of 0.35 to 1.00 (mean = 0.67, median = 0.69). The median value for the kappa score was equivalent to “substantial agreement” on the Landis and Koch scale (see Methods), with the range encompassing “fair agreement” to “perfect agreement”. Specific results of these analyses are available on request.

Approximately one third (34.1%; *n* = 108) of the media items were opinion pieces, while two-thirds were standard news stories (*n* = 209). The primary theme of the majority of media items (67.8%; *n* = 215,) was classified as “environmental”, while 51 items (16.1%) were “health” focused, and 33 items (10.4%) had both a health and environmental focus. Eighteen media items (5.7%) had another primary focus. In total, 95 media items related nitrates in drinking water to a potential health hazard (Table 1).

**Table 1:** Potential health hazards linked to nitrates in drinking water and frequency of mention in five years of media items (*n* = 317)

Potential health hazards specified	Frequency^a^	%
Colorectal cancer (or “bowel cancer”)	46	14.5
Methemoglobinemia (or “blue baby syndrome”)	23	7.3
Preterm birth	10	3.2
Birth defects (or “neural tube defects” or “congenital abnormalities”)	6	1.9
Other forms of cancer (or unspecified form of cancer)	6	1.9
Low birth weight	4	1.3
Cardiovascular disease^b^	1	0.3
Thyroid disease^b^	1	0.3

^a^Each health hazard was only counted once per media item. However, a media item could mention multiple health hazards or could relate nitrate in drinking water to a potential human health hazard but not actually specify the hazard

^b^The peer-reviewed literature suggests potential associations between nitrate ingestion and cardiovascular disease and thyroid disease [26]

For media items that related nitrates in water to one or more potential health hazards (*n* = 95; 30% of the total), we categorized the overall impression of the potential health hazard(s) and the level of uncertainty based on the judgement of the study team. However, in some cases there was insufficient information to categorize the media items. Overall, 6.3% (6/95) media items gave the overall impression that there was unlikely to be a health hazard, with the level of uncertainty expressed ranging from somewhat to highly uncertain. Over half (54.7%; 52/95) of the media items gave the overall impression that there was likely to be some health hazard, although the level of uncertainty expressed ranged from highly uncertain to highly certain (Table 2). Eight media items (8.4%) indicated that it was highly likely that there would be some health hazard, with the uncertainty ranging from somewhat to highly uncertain.

**Table 2:** Overall impression of potential health hazard for articles that mention colorectal cancer; methemoglobinemia; and preterm birth, low birth weight, or other adverse birth outcomes

Potential health hazard	Total # media items that mention health hazard^a^	Overall impression of potential health hazard			
		**not specified (%)**	**unlikely to be a health hazard (%)**	**likely to be some health hazard (%)**	**very likely to be some health hazard (%)**
**Colorectal cancer**	44	2 (4.5)	4 (9.1)	34 (77.3)	4 (9.1)
**Methemoglobinemia**	20	0	3 (15.0)	14 (70.0)	3 (15.0)
**Adverse birth outcomes (preterm birth, low birth weight, or other adverse outcomes)**	12	0	0	9 (75.0)	3 (25.0)

^a^Each health hazard was only counted once per media item. However, a media item could mention multiple health hazards or could relate nitrate in drinking water to a potential human health hazard but not actually specify the hazard

There was a strong emphasis on safe swimming in many media items in 2017, when the Government of the day changed the definition of “swimmable” waters. The primary health risk associated with swimming was the concentration of *E. coli* and waterborne pathogens. However, many articles solely focused on the ecological health of surface waters. There later became a stronger focus on the contamination of drinking water with nitrates, particularly in the Canterbury Region.

Overall, the total number of media items was highest in 2017 (*n* = 108, including 3 items for the period 17–31 December 2016)The fewest media items were identified in 2020 (*n* = 20). However, the percentage of media items with a health focus steadily increased over time, from 11.1% in 2017 to 51.2% in 2021 (Table 3).

**Table 3:** Frequency of media items with health focus and mention of specific health hazards^a^

Year	Total # media items	# with health focus^c^ (% of total media items)	# with health focus that mention colorectal cancer (% of total media items)	# with health focus that mention methemo-globinemia (% of total media items)	# with health focus that mention preterm birth, low birth weight, or other adverse birth outcomes (% of total media items)	# with health focus that mention other potential health hazards^d^ (% of total media items)
2017^b^	108	12 (11.1)	0	6 (5.6)	0	1 (0.9)
2018	47	7 (14.9)	2 (4.3)	1 (2.1)	0	0
2019	60	16 (26.7)	10 (16.7)	2 (3.3)	1 (1.7)	0
2020	20	7 (35.0)	3 (15.0)	1 (5.0)	0	0
2021	82	42 (51.2)	29 (35.4)	10 (12.2)	11 (13.4)	4 (4.9)
Total	317	84 (26.5)	44 (13.9)	20 (6.3)	12 (3.8)	5 (1.6)

^a^Media items can mention multiple health hazards or be health-focused but not specify a health hazard related to nitrates in water, therefore the sum of media items by specific health condition won’t necessarily equal the total number of health-focused media items

^b^Includes 3 items for the period 17–31 December 2016

^c^Includes items coded as either health focused or as having both a health and environmental focus

^d^Includes other cancers or unspecified forms of cancer

**Fig. 1:**
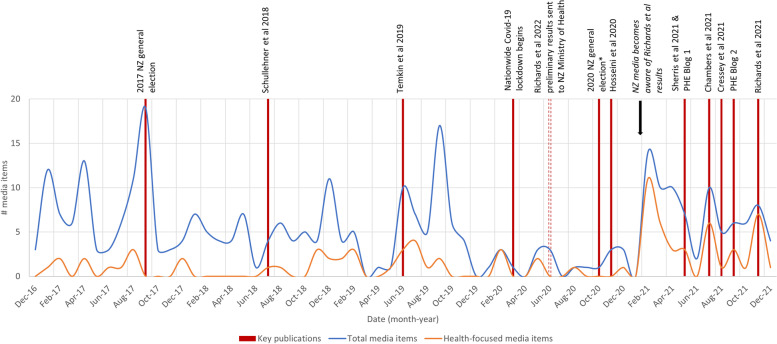
Timeline of scholarly publications linking to potential adverse health impacts of nitrates in drinking water, and other key events with the frequency of media items (see Supplementary Table 3 for additional details on key publications). *Debate prior to the 2020 NZ general election was dominated by Covid-19 issues, whereas water quality was a major issue in the 2017 NZ general election

The rise in the percentage of media items focused on the health impacts of nitrate contamination of water over time can be seen in Fig. 1 by the decreasing difference in the blue (total media items related to nitrates in freshwater captured by the search) and orange (media items focused on the health impacts of nitrate contamination of water extracted from the search) lines over time.

The majority of media items were generalisable to all of NZ (57.7%; *n* = 183), but many specifically focused on the Canterbury region (33.8%; *n* = 107) and frequently referred to waterbodies and catchments in that region (eg, Lake Ellesmere/Te Waihora, Ashburton River/Hakatere, Rakaia River, Waimakariri River, Selwyn River/Waikirkiri). Only 8.5% (*n* = 27) of the media items were focused on other regions of NZ (eg, Otago, Southland).

For media items with a health focus or an environmental and health focus (26.5%; *n* = 84), we tallied the first three stakeholders mentioned in the text, although 52 of the media items mentioned more than three stakeholders (Table 4). There were 93 mentions of political or governmental stakeholders (42.5% of all stakeholders), of which 54 (24.7%) were local government stakeholders and 39 (17.8%) were central government stakeholders. There were also 30 (13.7%) mentions of environmental or recreational stakeholders, 28 (12.8%) mentions of university researchers, 24 (11.0%) mentions of local residents or the general public, 18 (8.2%) mentions of agricultural or agriculture-adjacent (ie, irrigation companies) stakeholders, 16 (8.2%) mentions of international health organizations, two mentions of national health organizations, four mentions of researchers or scientists outside of a university setting, and one mention of iwi (Māori tribes).

**Table 4:** Types of stakeholders and frequency with which they were used as sources in health-focused or environmental and health focused media items (*n* = 84)^a^

Type of stakeholders	# of mentions	% of mentions
Local government	54	24.7
Central government	39	17.8
Environmental/recreational interest groups	30	13.7
University researchers	28	12.8
Local residents/general public	24	11.0
Agricultural interest groups	18	8.2
International health organizations	16	7.3
National health organisations^b^	2	0.9
Researchers/scientists outside university settings	4	1.8
Iwi or mana whenua (Indigenous people [Māori])	1	0.5
Other	3	1.4

^a^Some media items mention multiple stakeholders

^b^This category includes non-governmental health organizations (eg, includes Bowel Cancer NZ, but does not include the Ministry of Health, which is included in the “Central Government” category)

Notably, iwi or mana whenua (Indigenous people [Māori]) were only once one of the first three stakeholders mentioned in media items with a health or health and environmental focus, although in some cases they were mentioned later in media items. However, overall Māori values or perspectives were only mentioned in six (7.1%) of such media items.

We also collected stakeholders’ quotes that encapsulated their positions on potential health hazard(s) from the media items with a health focus or an environmental and health focus (26.5%, *n* = 84). Table 5 presents example quotes from different stakeholders by group. Collected quotes highlighted the strongly polarized views held by different stakeholder groups, as well as differences within certain stakeholder group (ie, governmental/political stakeholders, university researchers). However, there were minimal differences between quotes from stakeholders categorized as environmental/recreational stakeholders and local residents/general public. Preference was given to quotes that were longer and provided more details about stakeholders’ perspectives in the selection of example quotes for Table 5.

**Table 5:** Example quotes from health focused or health and environment focused media items

Position & Affiliation	Quote(s) from media items
***Governmental/Political Stakeholders***	
Deputy Director-General for the Ministry of Health	“[the Ministry deputy director-general] has previously said a ‘comprehensive review of the standards’ was under way, but the Danish study did not fully take into account other risks such as smoking, diet and obesity.”
Green Party Member of Parliament (MP)	“[the MP] said the studies showed the effects on human health happened at much lower levels than the World Health Organization standard for drinking water of 11.3 mg/L, which has influenced regional plan rules to ensure families avoided blue baby syndrome.”
Staff at the Ministry of Health	“Ministry of Health staff said they were happy with the current nitrate regulations and ignored calls for further study.”
Chief Scientist for Environment Canterbury (a local government agency)	Drinking water was “definitely” safe and would “continue to be so”…”quite a lot missing from a proper analysis and some pretty poor understanding”
Canterbury Medical Officer of Health	“midwives had become ‘the ambulance at the bottom of the cliff’ in parts of Canterbury, responsible for ensuring pregnant women did not drink water polluted with nitrates from their private bores.” “The implications if we don’t meet our targets—and we are not meeting our targets in Canterbury—is that we will see more sickness, we might see people dying… we’ve already seen people dying.” “Let’s not put our community at risk by exposing them to nitrates in their drinking water, let’s do the research… so we understand the real risk to New Zealanders.” “He told Stuff [news outlet] homes with nitrate levels above acceptable levels were not suitable for anyone to live in, not just pregnant women.”
***Environmental/Recreational Stakeholders***	
Chief Executive for Fish & Game	“…the cows are coming home to roost” “Some detractors will say this is scaremongering. It is not.”
Spokesperson for Aotearoa Water Action	“[the spokesperson] said the group was concerned about the lack of public notification on a consent that could have grave ramifications for human health and the environment. “
Chairperson for the Council of Outdoor Recreation Associations	“…no tangible action seems evident on rivers. For example the Canterbury Medical Officer of Health has warned of nitrate levels in the region’s water and its link to bowel cancer rates, extremely high in the region. The Government has dismissed the need for research.”
Secretary of the NZ Federation of Freshwater Anglers	“The NZ Federation of Freshwater Anglers has measured nitrate levels over recent years and the findings are alarming—toxic to trout, salmon smolt and native fish, and also well above safe human health levels. A 2019 Danish study of 2.7 million people reported a strong link between nitrate in drinking water and the risk of developing colorectal (bowel) cancer. Significantly, NZ has one of the highest bowel cancer rates in the world.”
Senior campaigner for Greenpeace	“Everyone should be able to trust that their drinking water is safe to drink but many of the people we have talked to today have been shocked and worried at the amount of nitrates in their drinking water” “In fact 100% of them [water samples] were over the 5 mg/l which is the risk for pre-term birth and all of them are over the cancer risk limit of 0.87 mg/l” “[the senior campaigner] pointed the finger at industrial dairy farming for allegedly ‘poisoning’ public and private water sources for profit.” “[the senior campaigner] said a precautionary approach was needed given emerging research showing links between nitrate contamination and human health effects, including bowel cancer and premature births.” “[the senior campaigner] said the 11.3 mg/L limit was for one health condition only—blue baby syndrome—and given the mounting evidence there was no guarantee even water under that level was safe”
***University researchers***	
Freshwater ecologist at Massey University, subsequently Victoria University	“…surface water in many parts of the land is highly contaminated with nitrates due to intensified farming. This is damaging freshwater ecosystems and biodiversity, and may harm human health.” “…this was an opportunity to make it more real for people to think about what is happening to their water in terms of human health.” “We would be failing in our duty if we hadn’t shared all this information with the people who are affected.” “…the US figures showed what a drain high nitrate levels could be on individuals’ health and on the economy”
Senior Research Fellow at the University of Otago, Wellington	“ ‘Those [obesity, smoking, alcohol consumption, fruit and vegetable consumption, physical activity] are the five major risk factors for colorectal (bowel) cancer that are known, and Southern, at a regional level, performs really well,’ [the Senior Research Fellow] said. ‘While that doesn’t say that nitrates is the reason why they’ve got higher rates of colorectal cancer, it does show that there is something else going on here.’ “ “[the Senior Research Fellow] said the limits in place now were set on the risk of blue baby syndrome. However, that limit did not consider any other potential health implications.” “Around 3.7% of all southern colorectal cancer or bowel cancer cases could be attributable to nitrate” “This research highlights the potential burden of nitrate contamination, particularly if further evidence on the link between nitrate and bowel cancer reinforces existing studies” “The results support the need to take a precautionary approach towards nitrate contamination in NZ” “…the authors [including the Senior Research Fellow] called for better reporting of nitrate levels in NZ and further research into the link between nitrates and adverse birth outcomes.”
Professor of Public Health at the University of Otago, Wellington	“…[they] have raised the alarming point that some water supplies in NZ, particularly in rural areas, have nitrate levels above the risky level in the Danish study.” “Another problem, he said, was that the report didn’t address the mounting problem of nitrates in water—something linked to colorectal cancer and other health risks.” “there has been increasing knowledge that low and intermediate levels of nitrates in drinking water are associated with adverse health outcomes.”
Postdoctoral Fellow at the Liggins Institute, University of Auckland	“There’s no conclusive evidence that nitrate in drinking water causes adverse birth outcomes such as preterm birth or birth defects. That’s according to my analysis of a decade’s research. But we can’t be complacent.”
***Local residents/general public***	
Canterbury resident	“My daughter was told not to drink Ashburton water while pregnant due to high nitrate levels.”
Springston resident (town in rural Canterbury)	“…the presenter outlined the very real threat of young babies developing methaemoglobinemia (blue baby syndrome) from high nitrates in Selwyn drinking water. This is a serious and potentially fatal illness. This seems like a very, very dangerous situation to me.” “…the huge health risks this will bring to the community. Expensive mitigations will be needed to ‘manage’ this massive increase in nitrate—from providing ‘safe’ water to young families…” “Now that valid scientific studies suggest a link between nitrate in drinking water and colorectal cancer, the stakes are raised even higher, and as a precaution, for the sake of my health and that of my family, I’m sourcing cleaner water. I would have thought BCNZ [Bowel Cancer NZ] would support the precautionary principle on this one.”
Strowan resident (suburb of Christchurch, Canterbury)	“Now there is real concern for the pollution of aquifer drinking water by nitrates, with the possibility of increasing the already high risk of bowel cancer.”
Resident of Maraetai (suburb of greater Auckland)	“Nitrates are beginning to find their way into the water table and aquifers. Currently this is not monitored. If these appear in drinking water there will be severe public health consequences.”
Resident of Riversdale (town in rural Southland)	“I think it’s very related to the high incidence of cancer in this area.”
***Agricultural sector***	
North Island Policy Manager for Federated Farmers	“There’s no link. There’s no causation there either.”
***Health entities***	
Medical advisor for Bowel Cancer NZ (a NGO)	“[the medical advisor] said when analysis of all seven studies was measured against the number of bowel cancer cases recorded, it was questionable that there was ‘anything there at all’.”
WHO	“The WHO responded that while it was possible ingesting nitrates could cause cancer in humans, other studies had shown conflicting data. It concluded the Danish study did not establish nitrate in drinking water caused colorectal cancer.”
***Iwi or mana whenua***	
Ngai Tuahuriri (an iwi [tribal] authority)	Regarding the Waimakariri committee’s proposal for nitrate levels: “Ngai Tuahuriri had also made clear it did not support aspects of the proposal”
***Other***	
Editor-at-large for Healthy Food Guide	“Until we know more about our levels of exposure to nitrates in water, there’s no reason to give it [drinking water] up.”

## Discussion

### Main findings and interpretation

Media items in NZ’s major newspapers were examined to assess the nature of the media discourse and determine how the release of journal articles, reports and scholarly blogs have influenced subsequent media discourse on nitrates in drinking water..

The temporal pattern of media items suggests that events of national importance (ie, the 2017 NZ general election) and the release of certain studies and scholarly blogs were associated with the publication of subsequent media items. There was a large increase in the number of media items published directly prior to the 2017 NZ general election, for which water quality was a major campaign issue. There also appeared to be an increase in the number of media items following the publication of the meta-analysis by Temkin et al. [13], which observed a statistically significant positive association for nitrate exposure from drinking water and adverse birth outcomes and colorectal cancer risk. Increases in the frequency of media items were also observed on several occasions in 2021 (Fig. 1), especially after the NZ media became aware of the preliminary results of a study by Richards et al. [4], which estimated that 3.26% (95%CI: 0.84, 5.57) of colorectal cancer cases in NZ were attributable to nitrates, resulting in 100 cases (95%CI: 25.7, 171.3) and 41 deaths (95%CI: 10.5, 69.7) annually.

There were also increases in media items that coincided with the publication of (i) a Letter to the Editor by Chambers et al. [27], which outlined concerns related to the meta-analysis by Hosseini et al. [28] and its conclusions on the quantification of the link between nitrates in drinking water and colorectal cancer; (ii) the release of a Public Health Expert (PHE) scholarly blog [29] published in response to a report co-funded by the major dairy company Fonterra and the NZ Ministry of Business Innovation and Employment (MBIE), which concluded that it is highly unlikely that nitrates in drinking water or diet present an increased risk of cancer [30]; and (iii) the publication of the study by Richards et al. [4] in *Environmental Research*.

The fewest media items were identified in 2020, likely to be partly due to the predominance of Covid-19 pandemic coverage that year. However, the percentage of media items with a health focus steadily increased over time, and there became a stronger focus on the contamination of drinking water with nitrates, particularly in the Canterbury Region, which has experienced rapid intensification of dairy farming and which typically uses extracted groundwater for drinking water.. The suggestive temporal association between the frequency of media items and the timing of key publications (ie, journal articles, reports, and scholarly blogs) presenting new research related to nitrates and human health could benefit from further investigation, possibly through a time series analysis of media items over a longer time period.

Generally, there seems to have been useful input from researchers (especially university-affiliated researchers) into the media discourse on nitrates in water. However, there has been relatively limited input from health officials, especially those at the national level, who often downplayed any potential risks (Table 5). Currently, the official Ministry of Health website on nitrate in drinking water only mentions methemoglobinemia as a potential health hazard [31]. Likewise, public statements from the Associate Minister of Health have focused on existing literature linking nitrate and colorectal cancer as not proving causation [32].

Pro-industry (agricultural) opinion pieces appear to be adequately balanced by opinion pieces by those concerned with public health, ecological health, and the values of recreational water users (eg, swimmers, anglers). However, there are multiple examples of the lines between agricultural spokespeople and researchers becoming blurred. For example, a report co-funded by Fonterra (the largest dairy company in NZ) and the Government concluded it was highly unlikely nitrate could contribute to bowel cancer [30]. However, this was based on an analysis with important limitations [29, 30]. Additionally, university researchers who were advising key health-related non-governmental organizations (NGOs) and acting on their behalf, have also contributed to a trade magazine for Ravensdown (the largest fertilizer company in NZ) [33]. Other researchers who have contributed to the media discourse while presenting themselves as independent, also sit on the boards of major agricultural companies and have failed to provide appropriate conflict of interest statements [34].

The media occasionally gave voice to poorly informed views (eg, stakeholders blaming improbable sources for water quality issues), and it is unclear whether there is sufficient skepticism by the media on pro-industry statements given the inherent competing commercial interests. Of particular concern, Māori perspectives were often neglected in health-focused media items. In addition, many media items tended to be one dimensional in their presentation of issues related to nitrates in water. For example, many health focused articles did not discuss the other dimensions of environment, recreational use, and cultural aspects of water quality protection. This may obscure the co-benefits of protecting freshwater from nitrate contamination.

### Study strengths and limitations

This study presents the first media discourse analyses of the issue of nitrates in water outside of Europe (to the best of our knowledge). Two previous studies, both conducted in Germany, have presented the results of discourse network analyses on the case of nitrate pollution of groundwater. Vogeler et al. [20] reported that clear sectoral divisions were visible in a region of Germany with intensive livestock farming, with an agrarian coalition that included individual farmers, farmers’ interest groups, municipalities that relied heavily on the agricultural sector, and certain political parties; and an environmental coalition that included environmental organizations and initiatives at the local level, as well as administrations responsible for environmental protection, and certain political parties (eg, Green Party). Schaub et al. [21] analyzed newspaper articles and press releases from 2010 to 2020 in Germany, and the results also indicated that two opposing stakeholder coalitions had formed over the issue of fertilizer regulation, and that the coalition in favor of tighter fertilizer regulation had highlighted the potential public health risks associated with contaminated drinking water. In this present study, strongly polarized views were also observed in the media discourse in NZ, a high-income nation with intensive dairy farming and other industrial agricultural production.

Limitations of this study include potential issues with content analysis coding, a relatively short time period, and focusing only on media items in major newspapers. Specifically, while there was good inter-observer reliability, it is still possible that there may have been some aspects of the discourse that were sub-optimally coded. Additionally, this study only examined media items for the five-year period from 17 December 2016 to 20 December 2021. Therefore, only three different central government administrations in NZ were captured, albeit two led by the same political party. Furthermore, we only searched for media items in major newspapers and may have missed important discourse that took place on radio, television, or social media platforms, or Māori and agricultural sector media. Further research that includes these other media formats would help to provide a more robust analysis of the discourse.

A further potential limitation of this study is that it has been conducted by a group of researchers who have actively contributed to the science outputs described here that have impacted the media discourse around nitrates, water, and human health in NZ. We do not consider this perspective has reduced the validity of our findings. It is a descriptive study, the methods used have been well described, and the analysis could be easily repeated by others.

### Potential implications for further research and more informed public discourse

As noted above, further analysis of different types of media for a longer time period (eg, 10 years), along with interviews with journalists and key spokespeople would help to provide a more robust view of the media discourse in NZ. Additionally, further investigation of the potential link between the release of new research and the timing of media items is warranted, possibly through a time series analysis. Investigation of how media coverage of public health issues influences decision making is also needed, although it was beyond the scope of this study. Such work could be justified given the importance of risk communication in communities that could be impacted by the contamination of drinking water with nitrates.

These issues are also relevant for the handling of other environmental health risks. Some environmental hazards result in unequivocal public health harm, such as enteric pathogen contamination of drinking water. However, the implications of other environmental contaminants, such as nitrates in drinking water, that are likely to have negative long-term effects, are more contested. This is where concepts such as the ‘precautionary principle’ are often proposed to prioritize the protection of public health in situations where the evidence is uncertain and management of risk can only be over a long period of time. An effort to discuss the co-benefits of protecting freshwater from nitrate contamination in the media could encourage the adoption of the precautionary principle by regulators and policy makers.

There are potential implications for policy making. While a diverse range of opinions were evident in this discourse analysis, often the views presented were very divisive (eg, farmers vs environmentalists) and it is possible that domains of consensus may have been overlooked in the media. Consequently, policy makers may be given the impression that they need to balance deeply polarized views that are not necessarily representative of public opinion. Therefore, as part of the policy making process it may be useful to pair a period of media discourse with a short period of a Polis-like process, as is used in Taiwan [35]. Polis has been directly used as part of policy making at both the national and local levels [35]. This type of process uses a pro-social media platform which builds domains of consensus so that policy makers can see normalized positions upon which to subsequently build policy solutions. For example, if such a process was used to help set new regulatory levels for nitrates in NZ, it might more clearly show politicians where New Zealanders stand on the issue of water quality. Nevertheless, previous surveys have suggested that the NZ public are generally very united in their views on freshwater and both urban and rural New Zealanders are equally concerned and identify agriculture as the primary cause of water pollution [36], despite media suggestions of a deep divide between urban and rural NZ.

## Conclusions

In summary, analysis of major newspapers for a five-year period provided a wide range of opinions and expertise to the public on the issue of nitrates in water. The percentage of media items with a health focus steadily increased over time, and the most commonly mentioned health hazard was colorectal cancer, followed by methemoglobinemia. The temporal pattern of media items suggests that the release of certain studies and scholarly blogs was associated with the publication of subsequent media items, although further research is required to confirm such a link. Governmental and political stakeholders were the most frequently mentioned in media items, while Māori values and perspectives were rarely acknowledged. Many media items only discussed a single dimension of the issue of nitrates in water, while neglecting other dimensions (eg, health, environmental, recreational, and cultural aspects). Further analysis of different types of media for a longer time period (eg, 10 years) would help to provide a more robust view of the media discourse in NZ and elucidate the potential link between the release of new research and the timing of media items.

## Supplementary Information

**Additional file 1: Supplementary Table 1.** NZ newspaper readership in 2021^1^ [37].

**Additional file 2: Supplementary Table 2.** Content analysis coding schedule.

**Additional file 3: Supplementary Table 3.** Timeline of release of key publications during the five-year period of media item analysis in this study.

## Data Availability

The data generated and analyzed during the current study are available from the corresponding author on reasonable request.

## Abbreviations

NZ: New Zealand
WHO: World Health Organization
MBIE: Ministry of Business, Innovation and Employment
PHE: Public Health Expert (Blog)
