# Supplementary material for: The potential human health hazard of nitrates in drinking water: a media discourse analysis in a high-income country
Source: Environ Health. 2023 Jan 20;22:9. doi: 10.1186/s12940-023-00960-5 (PMC9851889; doi:10.1186/s12940-023-00960-5)
Supplement: Supplementary file 1 — Additional file 1: Supplementary Table 1. NZ newspaper readership in 20211 [37]. [file 12940_2023_960_MOESM1_ESM.docx]

**Supplementary Table 1. NZ newspaper readership in 2021^1^ (37)**

| **Newspaper** | **Readership^2^** |
| --- | --- |
| New Zealand Herald | 1,844,000 |
| Dominion Post | 428,000 |
| The Press | 293,000 |
| Otago Daily Times | 285,000 |
| Sunday Star-Times | 230,000 |
| ^1^Fiscal year ending June 2021  ^2^Total cross-platform audience (print, internet, or app) | |
