# Supplementary material for: The potential human health hazard of nitrates in drinking water: a media discourse analysis in a high-income country
Source: Environ Health. 2023 Jan 20;22:9. doi: 10.1186/s12940-023-00960-5 (PMC9851889; doi:10.1186/s12940-023-00960-5)
Supplement: Supplementary file 2 — Additional file 2: Supplementary Table 2. Content analysis coding schedule. [file 12940_2023_960_MOESM2_ESM.docx]

**Supplementary Table 2. Content analysis coding schedule**

Included media items were subjected to a content analysis coding for the following:

| Unique media item identifier |
| --- |
| Title |
| Date of publication |
| Newspaper |
| Syndication to another media item |
| Author(s) |
| Type of media item |
| Categorization of the primary theme |
| Categorization of the potential health hazard(s) |
| Categorization of the overall impression of the potential health hazard(s) on a scale: unlikely to be a health hazard, likely to be some health hazard, or very likely to be some health hazard |
| Categorization of the overall impression of uncertainty around the level of potential health hazard(s) on a scale: highly uncertain, somewhat uncertain, highly certain |
| Main water source discussed |
| Specific waterbodies mentioned |
| Mention of recreational water use |
| Mention of the impact of water pollution on the environment |
| Mention of Māori values or perspectives |
| Stakeholders |
| Quotes from stakeholders encapsulating position on potential health hazard(s) |
| Mention of any journal articles, reports, or scholarly blogs |
