# Supplementary material for: The potential human health hazard of nitrates in drinking water: a media discourse analysis in a high-income country
Source: Environ Health. 2023 Jan 20;22:9. doi: 10.1186/s12940-023-00960-5 (PMC9851889; doi:10.1186/s12940-023-00960-5)
Supplement: Supplementary file 3 — Additional file 3: Supplementary Table 3. Timeline of release of key publications during the five-year period of media item analysis in this study. [file 12940_2023_960_MOESM3_ESM.docx]

**Supplementary Table 3. Timeline of release of key publications during the five-year period of media item analysis in this study**

| **Date** | **First Author** | **Title** | **Details** |
| --- | --- | --- | --- |
| 1 Jul 2018 | Schullehner | Nitrate in drinking water and colorectal cancer risk: A nationwide population‐based cohort study | Danish cohort study linking nitrates in drinking water to colorectal cancer published in a the *International Journal of Cancer* |
| 11 Jun 2019 | Temkin | Exposure-based assessment and economic valuation of adverse birth outcomes and cancer risk due to nitrate in United States drinking water | Meta-analysis of adverse birth outcomes and nitrate in US drinking water becomes available online at the website for the journal *Environmental Research* |
| Jun 2020 | Richards | Nitrate contamination in drinking water and colorectal cancer: Exposure assessment and estimated health burden in New Zealand | Preliminary results submitted to NZ Ministry of Health |
| 9 Nov 2020 | Hosseini | Nitrate-nitrite exposure through drinking water and diet and risk of colorectal cancer: A systematic review and meta-analysis of observational studies | Factually incorrect meta-analysis of nitrate in drinking water and diet and colorectal cancer risk accepted by the journal *Clinical Nutrition* |
| 5 May 2021 | Sherris | Nitrate in Drinking Water during Pregnancy and Spontaneous Preterm Birth: A Retrospective Within-Mother Analysis in California | Analysis of nitrate in drinking water and preterm birth in California published in the journal *Environmental Health Perspectives* |
| 24 May 2021 | Chambers | Nitrate contamination in drinking water and adverse birth outcomes: emerging evidence is concerning for NZ | Public Health Expert (PHE) scholarly blog released |
| 12 Jul 2021 | Chambers | Letter to the editor: Correction “Nitrate-nitrite exposure through drinking water and diet and risk of colorectal cancer: A systematic review and meta-analysis of observational studies” | Letter to the editor regarding issues with Hosseini et al (2020) published in the journal *Clinical Nutrition* |
| 13 Aug 2021 | Cressey | Nitrate in food and water | Report co-funded by the major NZ dairy company Fonterra and NZ Ministry for Business, Innovation and Employment (MBIE) concluded "it is highly unlikely that nitrates in drinking water or diet present an increased risk of cancer" published |
| 3 Sep 2021 | Chambers | Public health response to report on potential risk of nitrate from drinking water | PHE scholarly blog released |
| 2 Nov 2021 | Richards | Nitrate contamination in drinking water and colorectal cancer: Exposure assessment and estimated health burden in New Zealand | Journal article becomes available online (published in the journal *Environmental Research*) |
